# Development of Film Dosage Form Containing Allopurinol for Prevention and Treatment of Oral Mucositis

**DOI:** 10.5402/2012/764510

**Published:** 2012-03-15

**Authors:** Yoshifumi Murata, Kyoko Kofuji, Norihisa Nishida, Ryosei Kamaguchi

**Affiliations:** ^1^Faculty of Pharmaceutical Science, Hokuriku University, Ho-3, Kanagawa-machi, Kanazawa 920-1181, Japan; ^2^Morishita Jintan Co. Osaka Technocenter, 2-11-1, Tudayamate, Hirakata, Osaka 573-0128, Japan

## Abstract

Film dosage forms (FDs) containing allopurinol (AP) were prepared using a casting method with water-soluble polysaccharides, such as sodium alginate (ALG), and the release profile of AP from FDs was investigated in limited dissolution medium. Some ALGs were able to form FDs incorporating AP, and the thickness was about 50 **μ**m. All FDs were easy to handle, though the rheological properties varied with ALG species. AP was homogenously present throughout the FDs and was released with disintegration in 10 mL of physiological saline. These results confirmed that FDs are useful for preventing or treating localized problems in the oral cavity, such as mucositis. FDs are also useful for administering drugs to cancer patients receiving chemotherapy and/or radiotherapy.

## 1. Introduction

The xanthine oxidase inhibitor allopurinol (AP) is a drug used for the treatment of gout. AP has also been used to prevent and treat oral mucositis (mouth ulcers) in cancer patients receiving chemotherapy and/or radiotherapy [1–3]. In the treatment of mucositis, direct application of AP to the oral mucosa is necessary to avoid the systemic action seen after gastrointestinal absorption as the antioxidant activity of AP will result in lowering the activity of anticancer drug such as methotrexate. For example, AP suspensions (1 mg/mL) are freshly prepared in the hospital, and the patient gargles with the preparation [4]. This method for preventing mucositis is simple and effective; however, these preparations are not stable for long-term preservation, and it is difficult to control the amount of AP during gargling, although the dose is not sufficiently high to induce systemic effects in the event of erroneous swallowing.

Recently, oral disintegration (OD) dosage forms such as OD tablets have become widely utilized, as they are useful in patients who have difficulty swallowing regular tablets [5, 6]. Film dosage forms (FDs) are also anticipated to be useful in oral care [7–9]. The active compound present in FDs is spread in the oral cavity as the form disintegrates on contact with saliva, and the disintegration profile of FDs can be varied by modifying the film base [10]. However, the drug loading capacity of FDs is typically very low, and the drug incorporated into FDs should be selected carefully.

FDs are generally prepared using a water-soluble polymer base for immediate dissolution in saliva. As various polysaccharides have been used as additives for drug preparation or food ingredients because of the safety on peroral administration, they are candidate materials for FD preparation. Algal polysaccharide sodium alginate (ALG) consists of *α*-L-guluronate and *β*-D-mannuronate and is able to form thin films [11, 12]. Pullulan (PUL), a polysaccharide composed of *α*-D-maltotriose, is also a known film base [13]. In this study, we prepared FDs containing AP using a casting method with natural polysaccharides without dissolution in organic solvents, heating, pH regulation, or addition of plasticizer. The release profiles for AP from FDs were then investigated in limited dissolution medium, as AP is expected to be active following dissolution in saliva upon oral FD administration.

## 2. Materials and Methods

### 2.1. Materials

Three species of ALG (300 cps, 500 cps, and 1000 cps) were obtained from the Nacalai Tesque Inc. (Kyoto, Japan). Low-molecular-weight alginate (L-ALG) was obtained from Alfa Aesar (Ward Hill, MA, USA). Guluronic-acid-rich alginate (20 G) was supplied by the Kibun Food Chemifa Co. (Tokyo, Japan). PUL was supplied by the Hayashibara Biochemical Laboratories (Okayama, Japan). Polygalacturonic acid (PGA) was purchased from the MP Biomedicals (Solon, OH, USA). AP was purchased from the Wako Pure Chemicals (Osaka, Japan). All other chemicals were of reagent grade.

### 2.2. FD Preparation

Polysaccharides were dissolved in deionized water with agitation, and 1.5–4% (W/W) solutions were prepared. AP (10 mg) was added to 10 g of the film base solutions, followed by thorough mixing, and 3.0 g of each solution was poured into a plastic Petri dish (diameter, 54 mm). After 24 h at 37°C, the circular films formed on the dish were transferred to a desiccator. Film formation was judged to have failed if a film could not be removed from the bottom of the dish. Film surfaces were observed with a digital microscope (VHX-900; Keyence Co., Osaka, Japan).

### 2.3. Film Thickness and Rheological Properties

Film thickness was measured at 10 points on each film using a micrometer (CLM1-15QM; Mitutoyo, Kawasaki, Japan) with a set pressure of 0.5 N. Measurements were made on 3 films, and the mean thickness was calculated for each type. The rheological properties of each film were determined using a rheometer (SUN RHEO TEX SD-700#; Sun Scientific Co., Tokyo, Japan) at room temperature. The film was fixed on a vial (inner diameter, 1.4 mm; outer diameter, 18.8 mm) using joining tape (Scotch mending tape; Sumitomo 3 M Ltd., Tokyo, Japan) and was probed with a cylindrical adapter (diameter, 5.0 mm). Stress and strain were measured at the point at which the adapter broke through the film, and tests were performed in triplicate.

### 2.4. X-Ray Diffractometry

X-ray diffractometry was carried out using an automatic diffractometer (D8 DISCOVER with GADDS; Bruker AXS K.K., Yokohama, Japan) with a voltage of 40 kV and a current of 40 mA. The results of X-ray diffraction were interpreted using computer software (Bruker AXS K.K.).

### 2.5. AP Dissolution Test

Physiological saline was used as the dissolution test medium. Films were placed in a plastic dish, and 10 mL of the dissolution medium preheated to 37°C was added. The dish was shaken at 300 rpm in a shaker incubator (SI-300; As One Co., Osaka, Japan) at 37°C. After 1, 3, 5, 10, 15, 20, 30, 45, and 60 minutes, 80 *μ*L aliquots were removed and placed into micro-test tubes (1.5 mL), and 720 *μ*L of methanol was added to precipitate the polysaccharide dissolved from the FD. Samples were mixed and centrifuged (7,700 × g, 5 min; H-1300; Kokusan Co., Saitama, Japan), and the supernatants were injected into an HPLC column. All tests were performed in triplicate. The HPLC system comprised an LC-6A pump (Shimadzu Co., Kyoto, Japan), a packed column (150 mm × 4.6 mm, COSMOSIL 5C_18_-MS-II, Nacalai Tesque), and a SPD-6A UV detector (Shimadzu Co.). HPLC for the determination of AP was conducted at ambient temperature using 20 mM acetate buffer (pH 4.5) at a flow rate of 1.0 mL/min [14]. The detector wavelength was set at 254 nm.

## 3. Results and Discussion

**Figure 1:**
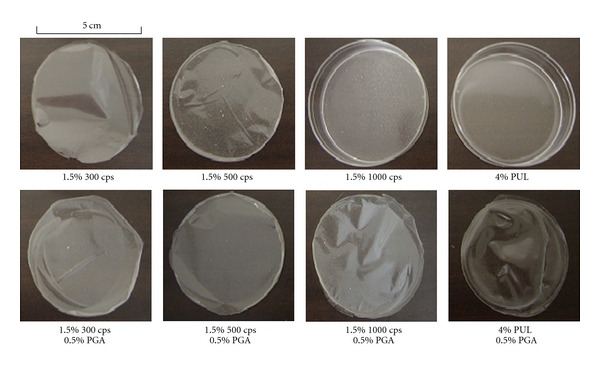
Images of FDs prepared with polysaccharides containing AP.

Using the casting method, 1.5–2% ALG or 4% PUL was poured into molds and the solvent was evaporated from the solution. The polysaccharides used in this study form circular films when the solution does not contain AP. In the presence of AP (1 mg/g), both 300 cps and 500 cps formed circular films, but 1000 cps and PUL did not form films, as shown in Figure 1. On the other hand, all of the polysaccharides were able to form films with the addition of 0.5% PGA to the film base solution, although cracks were observed in the case of films prepared with 4% PUL and 0.5% PGA. The thicknesses of FDs prepared with ALGs were 44–65 *μ*m, as shown in Table 1. L-ALG (2%) and 20 G (2%) also formed films, and the thicknesses were 48 ± 3 *μ*m and 37 ± 2 *μ*m, respectively. These results indicate that ALG is useful as a film base to prepare FDs incorporating AP.

**Figure 2:**
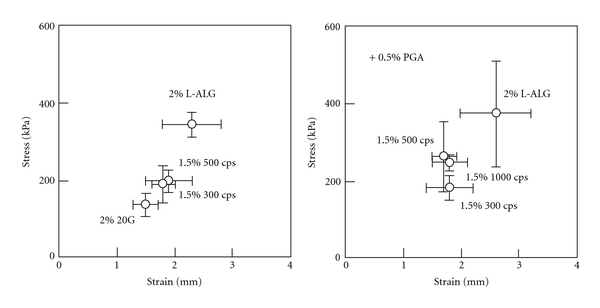
Rheological properties of alginate films containing AP (*n* = 3).

FDs containing AP are applied to the oral cavity; therefore, they must be easy to handle.  Figure 2 shows the rheological properties of FDs prepared with ALGs. All films were easy to handle and resistant to tearing, although FD properties varied with ALG species. Adding 0.5% PGA to the film base solution did not affect FD strength, and no apparent relationship between film thickness and hardness was observed.

**Figure 3:**
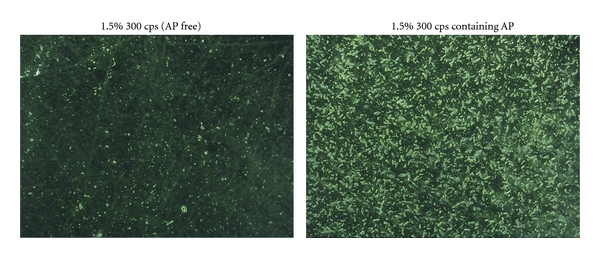
FD surface observed under digital microscope (×50).

AP is dissolved in the polysaccharide solution, and the drug is incorporated into the matrix gradually formed by the polymer base. As shown in Figure 3, AP is homogenously present throughout the film. Therefore, AP would be distributed across the region to which FD is applied in the oral cavity.

**Figure 4:**
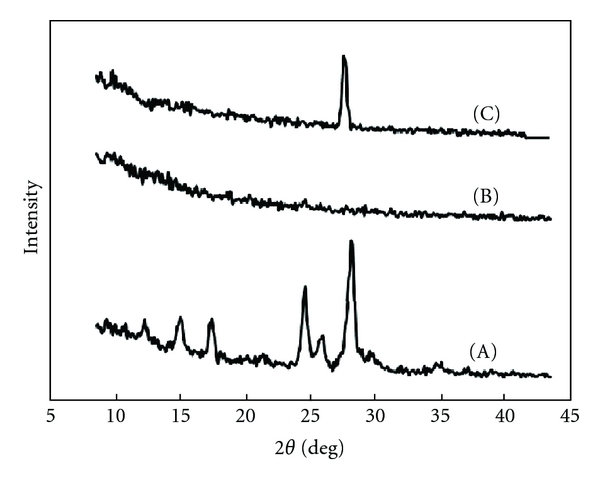
X-ray diffractograms. (A) AP (powder); (B) 1.5% 300 cps film (AP free); (C) 1.5% 300 cps film containing AP.

Figure 4 shows the X-ray diffraction patterns for AP powder and FDs prepared with 1.5% 300 cps. AP exhibited a characteristic crystalline compound diffraction pattern. On the other hand, the diffraction pattern of FD was similar to that of an amorphous polymer, and FD containing AP showed a different pattern than AP powder. These results indicate that the crystal form of AP is only slightly present in FDs.

**Figure 5:**
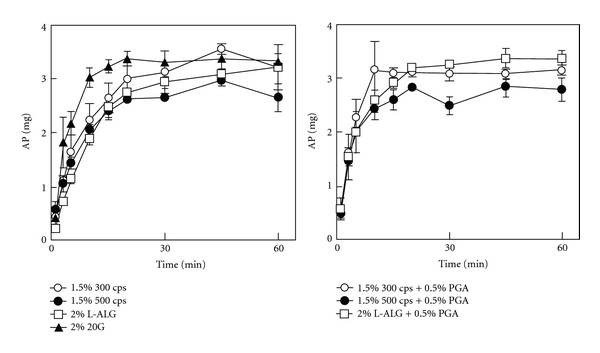
Release profiles of AP from FDs in physiological saline (*n* = 3).

In the treatment of mucositis, solutions or suspensions of AP are administered in the oral cavity to act directly at the inflammation site. Therefore, AP needs to be released immediately upon contact with saliva, which is secreted from the salivary glands at 1.5–2.0 mL/min after stimulation [15]. In this study, films were soaked in 10 mL of physiological saline, and the amount of AP released from the FDs was measured. In all FDs prepared with ALG, rapid swelling in the dissolution medium was observed with the naked eye, and the film itself dissolved in the medium within 20–30 min. As shown in Figure 5, incorporated AP was immediately released, irrespective of the ALG species used as a film base. In particular, all of the AP was released within 10 min from the FD prepared with 2% 20 G. Similar AP release profiles were obtained with FDs containing PGA. However, the preparation did not dissolve in physiological saline, and film residue remained at the end of the dissolution test. These results show that AP is present in the polymer matrix and is released through pores formed by permeation of the dissolution medium.

## 4. Conclusions

FDs allow the distribution of a drug across the region to which the FD is attached. In this study, FDs were prepared using natural polysaccharides without addition of plasticizers or dissolution into organic solvents. FDs prepared with ALG are able to incorporate AP and immediately release the drug in limited dissolution medium. FDs are thus useful for preventing or treating localized problems in the oral cavity, such as mucositis. They also simplify the administration of drugs to patients.

## Figures and Tables

**Table 1:** Thickness of FDs containing AP.

Film base	Thickness (*μ*m, *n* = 3)
1.5% 300 cps	44 ± 1
1.5% 500 cps	55 ± 9
1.5% 300 cps + 0.5% PGA	50 ± 3
1.5% 500 cps + 0.5% PGA	65 ± 12
1.5% 1000 cps + 0.5% PGA	48 ± 6
